# Sugar-binding and split domain combinations in repeats-in-toxin adhesins from *Vibrio cholerae* and *Aeromonas veronii* mediate cell-surface recognition and hemolytic activities

**DOI:** 10.1128/mbio.02291-23

**Published:** 2024-01-03

**Authors:** Mustafa Sherik, Robert Eves, Shuaiqi Guo, Cameron J. Lloyd, Karl E. Klose, Peter L. Davies

**Affiliations:** 1 Department of Biomedical and Molecular Sciences, Queen’s University, Kingston, Ontario, Canada; 2 South Texas Center for Emerging Infectious Diseases and Department of Molecular Microbiology and Immunology, University of Texas San Antonio, San Antonio, Texas, USA; University of Texas Health Science Center, School of Public Health, Houston, Texas, USA

**Keywords:** *Vibrio cholerae*, adhesins, enteric pathogens, hemolysis, glycan, calorimetry, protein modeling

## Abstract

Many pathogenic Gram-negative bacteria use repeats-in-toxin adhesins for colonization and biofilm formation. In the cholera agent *Vibrio cholerae*, flagellar-regulated hemagglutinin A (FrhA) enables these functions. Using bioinformatic analysis, a sugar-binding domain was identified in FrhA adjacent to a domain of unknown function. AlphaFold2 indicated the boundaries of both domains to be slightly shorter than previously predicted and assisted in the recognition of the unknown domain as a split immunoglobulin-like fold that can assist in projecting the sugar-binding domain toward its target. The AlphaFold2-predicted structure is in excellent agreement with the molecular envelope obtained from small-angle X-ray scattering analysis of a recombinant construct spanning the sugar-binding and unknown domains. This two-domain construct was probed by glycan micro-array screening and showed binding to mammalian fucosylated glycans, some of which are characteristic erythrocyte markers and intestinal cell epitopes. Isothermal titration calorimetry further showed the construct-bound l-fucose with a *K*_d_ of 21 µM. Strikingly, this recombinant protein construct bound and lysed erythrocytes in a concentration-dependent manner, and its hemolytic activity was blocked by the addition of l-fucose. A protein ortholog construct from *Aeromonas veronii* was also produced and showed a similar glycan-binding pattern, binding affinity, erythrocyte-binding, and hemolytic activities. As demonstrated here with Hep-2 cells, fucose-based inhibitors of this sugar-binding domain can potentially be developed to block colonization by *V. cholerae* and other pathogenic bacteria that share this adhesin domain.

**IMPORTANCE:**

The bacterium, *Vibrio cholerae*, which causes cholera, uses an adhesion protein to stick to human cells and begin the infection process. One part of this adhesin protein binds to a particular sugar, fucose, on the surface of the target cells. This binding can lead to colonization and killing of the cells by the bacteria. Adding l-fucose to the bacteria before they bind to the human cells can prevent attachment and has promise as a preventative drug to protect against cholera.

## INTRODUCTION

The initial attachment of bacteria to their preferred binding locations can be mediated through a variety of surface proteins (1). Those proteins that specialize in adhesion include the repeats-in-toxin (RTX) adhesins found in many Gram-negative bacteria (2, 3). They derive their name from the tandemly repeated, calcium-binding, nonapeptide RTX sequences first described in secreted hemolysins and toxins (4, 5). These glycine- and aspartate-rich repeats form a characteristic β-solenoid structure just upstream of the C-terminal type I secretion signal (6). Although the RTX adhesins also pass through the type I secretion system, they are retained in the outer bacterial membrane by their N-terminal domain, which plugs the export channel (7, 8). RTX adhesins have one or more ligand-binding domains just upstream of the RTX domain and a variable number of immunoglobulin-like β-sandwich domains that extend the ligand-binding domain away from the bacterial surface (3).

The first structurally characterized RTX adhesin was described in a marine bacterium, *Marinomonas primoryensis*, isolated from an ice-covered lake in Antarctica (9). This 1.5-MDa protein contains three ligand-binding domains adjacent to the RTX repeats. The most distal domain is an ice-binding protein that alone is responsible for attaching the bacterium to the underside of the lake ice cover (10). Next is a PA14 sugar-binding domain with a preference for binding fucose-tipped glycans (11), followed by a peptide-binding domain (PBD) that attaches to the C-terminal three residues of proteins (12). The PBD shows distinct sequence preferences that range over a 1,000-fold difference in binding affinity. The fucose- and peptide-binding domains attach the motile *M. primoryensis* to the non-motile diatom *Chaetoceros neogracile* and anchor this mixed microorganism colony to the underside of lake ice in a mutually beneficial arrangement (9).

RTX adhesins are widespread in Gram-negative bacteria including many human pathogens like *Aeromonas veronii*, *Vibrio cholerae*, and other members of the *Vibrio* genus (3). *A. veronii* is a natural symbiont of *Hirudo verbena* leeches, where it resides in the gastrointestinal tract to assist the organism digesting blood (13). In humans, *A. veronii* can cause diseases ranging from wound infections and diarrhea to sepsis. This bacterium is equipped with a 0.613-MDa RTX adhesin although its role in pathogenesis is poorly understood (12). *V. cholerae* O1 strains, which have caused multiple cholera pandemics, are equipped with an RTX adhesin known as flagellar-regulated hemagglutinin (FrhA) that is responsible for both attachment to organisms in the marine environment and for enhancing intestinal colonization (14). *V. cholerae* pandemic strains colonize the human gastrointestinal tract following the ingestion of contaminated food or water, and express cholera toxin, which leads to the characteristic diarrhea that is the hallmark of the disease cholera (15, 16). *V. cholerae* pandemic strains also develop biofilms on various surfaces in the marine environment during inter-epidemic periods, including chitinous surfaces such as zooplankton (17–19). FrhA has been shown to enhance *V. cholerae* binding to human epithelial cells, gastrointestinal colonization, and biofilm formation on chitin and other surfaces (14, 20). FrhA is much smaller at 0.235 MDa than the *M. primoryensis* RTX adhesin because it has far fewer extender domains. At the distal end, there are two recognized ligand-binding domains. One is a PBD homolog with 70% sequence identity to the one in the *M. primoryensis* RTX adhesin (12). C-terminal of this is a putative sugar-binding domain (SBD) followed by a domain of unknown function (UKD).

Here, we have investigated the structure and function of SBD and its UKD neighbor. Modeling with AlphaFold2 and small-angle X-ray analysis indicated that these two domains are a structural unit with UKD serving as a “split domain” that supports SBD and projects it outward from the axis of the adhesin to meet its ligand. Glycan array analysis of SBD-UKD from *V. cholerae* and its homolog from *A. veronii* showed binding to human fucose-tipped major blood group antigens found on red-blood cells and other cell types in the body. Fluorescently labeled *V. cholerae* SBD-UKD bound and lysed human erythrocytes in a concentration-dependent manner. Both binding and lysis were blocked by low levels of free fucose.

## RESULTS

### The ligand-binding region of FrhA contains a sugar-binding domain

**Fig 1:**
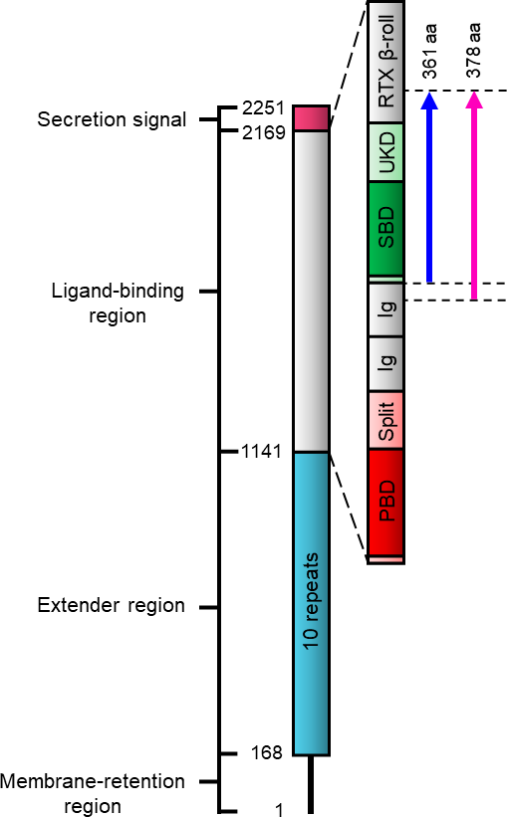
Domain map of FrhA. A schematic representation of the domain architecture of FrhA oriented from N to C terminus. Regions and domains predicted with high confidence are represented by rectangles, while regions predicted with low confidence are represented by a black horizontal line. Domains in the ligand-binding region are labeled as follows: PBD, peptide-binding domain; Split, split Ig-like domain; Ig, Ig-like domain; SBD, sugar-binding domain; UKD, unknown domain. The pink and blue arrows spanning sections of the ligand-binding region represent the *Vc*SBD-UKD and *Av*SBD-UKD constructs, respectively.

One of the difficulties of working with RTX-adhesins, which fold into long chains of domains, is knowing where one domain ends and another begins. Prior to the release of AlphaFold2 (21), a Phyre2 search was conducted on the ligand-binding region of *V. cholerae* FrhA (AWB74152.1) that identified a putative sugar-binding domain (SBD) and an adjacent domain of unknown function (UKD) in addition to other domains previously recognized in RTX adhesins (Fig. 1) (20). Both SBD and UKD were predicted to adopt anti-parallel β-sandwich structures although the structure of the UKD was predicted with low confidence (data not shown). It was reasoned that the UKD lay entirely between the SBD and RTX β-roll domains. Based on this modeling combined with protein BLAST searches and multiple sequence alignments, the SBD and UKD were thought to lie between residues 1677–1853 and 1854–2016, respectively. Attempts to express soluble SBD separate from the UKD have been unsuccessful. This observation has been made with other ligand-binding domains of RTX adhesins. It is possible that the SBD requires the UKD to properly fold into a stable construct. To study the SBD and UKD of FrhA, a His-tagged 42 kDa protein construct spanning both domains was designed.

A protein BLAST search was conducted using the combined *Vc*SBD-UKD sequence as a probe. High sequence identity was found to proteins in adhesins from other pathogenic bacteria such as *V. vulnificus* (>99%) and *A. veronii* (~70%). To confirm results of experiments involving *Vc*SBD-UKD, a 40 kDa ortholog construct from *A. veronii* was designed (WP_103422706.1). Of the ~70% sequence identity shared between *Av*SBD-UKD and *Vc*SBD-UKD, ~80% identity was attributed to the SBD and ~60% to the UKD (Fig. S1). Upon reexamination of *Vc*SBD-UKD’s protein sequence against sequence homologs of other bacterial species, it was noticed that the first 17 residues on the N-terminal side were the least conserved. It was reasoned these residues may not be critical for protein folding and, consequently, were omitted from the design of the *Av*SBD-UKD construct.

### Purified *Vc*SBD-UKD and *Av*SBD-UKD degrade to smaller more stable proteins

To purify *Vc*SBD-UKD, we initially used nickel-affinity chromatography (Ni-NTA), resulting in two distinct and highly enriched bands near the expected molecular weight of *Vc*SBD-UKD (Fig. S2A). The upper band, at approximately 42 kDa, exhibited slightly stronger intensity in comparison to the lower 37 kDa band. On the assumption that the smaller band was derived from the larger one by endogenous proteolysis, a sample of the Ni-NTA elution fraction was reapplied to the Ni-NTA column to check for retention on the column. Both bands were well retained on the column and eluted by imidazole (data not shown). Since the His-tag was at the N terminus, this implied any shortening was due to proteolysis at the C-terminal end. A sample from the Ni-NTA elution was subjected to limited proteolysis to assess fragment stability. After a month at 4°C, the lower molecular weight band was the only remaining species (Fig. S3). Efforts to separate the two bands from each other using size-exclusion chromatography (Fig S2B and C) were attempted but proved unsuccessful. The SEC chromatogram depicted a single broad elution peak within the expected molecular weight range of *Vc*SBD-UKD construct.

For *Av*SBD-UKD, Ni-NTA produced two protein bands near the expected molecular weights seen for the purification of *Vc*SBD-UKD (Sup. 4A). Unlike *Vc*SBD-UKD though, the lower band was more intense than the upper band in the Ni-NTA elution. To separate the protein species from one another, anion-exchange chromatography was used. The resulting chromatogram showed two distinct peaks, with the lower band eluting first at a lower salt gradient, while the higher band eluting second at a higher salt gradient (Fig. S4B and C).

### *Vc*SBD-UKD and *Av*SBD-UKD bind to fucosylated glycans

**Fig 2:**
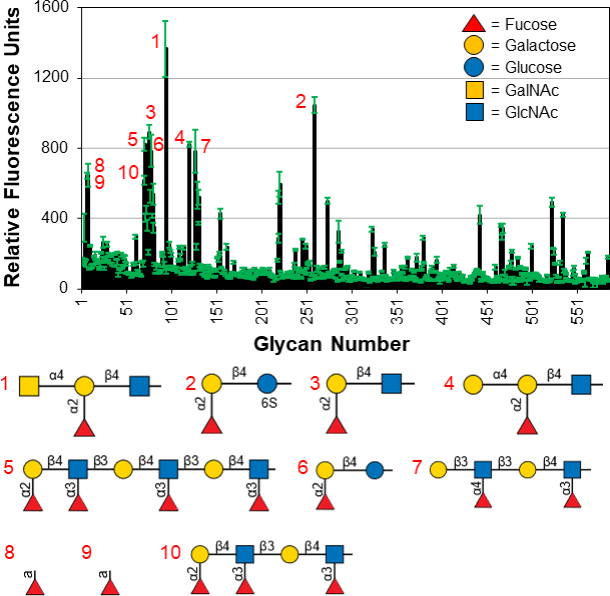
Glycan array results for fluorescently labeled *Vc*SBD-UKD. Glycan array data for *Vc*SBD-UKD are represented in relative fluorescence units with the standard deviation of four replicates indicated by green error bars. The top 10 glycan binders from the data set are indicated by number and shown in a schematic representation below the plot ranked from highest to lowest by signal strength. A legend describing the different sugar units is embedded in the plot.

To determine the ligand specificities of the two SBD-UKD constructs, we fluorescently labeled the purified proteins and screened them against mammalian glycan chips. Data from an array containing 585 unique glycans indicated that *Vc*SBD-UKD preferred to bind fucosylated glycans (Fig. 2). The top 10 hits consisted of glycans with α(1, 2), α(1, 3), and α(1, 4) fucose linkages. A few examples include GalNAcα1–4(Fucα1–2)Galβ1–4GlcNAcβ-Sp8, Fucα-Sp9, Fucα1–3GlcNAcβ-Sp8, and Galβ1–3(Fucα1–4)GlcNAcβ1–3Galβ1–4(Fucα1–3)GlcNAcβ-Sp0. The corresponding relative fluorescence unit (RFU) measurements for these four glycans were 1,366, 644, 522, and 785, respectively. On average, higher RFU measurements were observed for α(1, 2) fucose linkages to galactose than the other types of linkages although many glycans with α(1, 2) fucose linkages had extremely low RFU values. These examples include Fucα1–2Galβ1–3GlcNAcβ1–6(Fucα1–2Galβ1–3GlcNAcβ1–3)GalNAcα-Sp14 and Fucα1–2Galβ1–3GalNAcα1–3(Fucα1–2)Galβ1–4Glcβ-Sp0. Glycans with fucose α(1,6) linkages bound with the lowest RFU compared to the other types of linkages.

**Fig 3:**
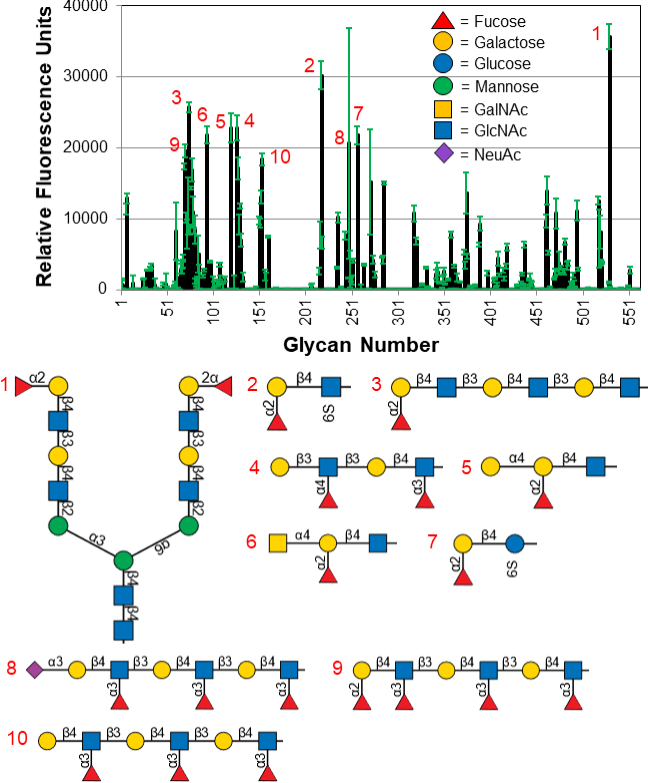
Glycan array results for GFP-tagged *Av*SBD-UKD. Glycan array data for *Av*SBD-UKD are represented in relative fluorescence units with the standard deviation of four replicates indicated by green error bars. The top 10 glycan binders from the data set are indicated by number and shown in a schematic representation below the plot ranked from highest to lowest by signal strength. A legend describing the different sugar units is embedded in the plot.

To test if *Av*SBD-UKD has a similar binding specificity to *Vc*SBD-UKD, GFP-tagged *Av*SBD was probed against a glycan chip that contained 561 mammalian glycans. Screening results for *Av*SBD-UKD also indicated specificity toward fucosylated glycans (Fig. 3). The glycan array screening data for *Av*SBD-UKD displayed higher RFU values than the data for *Vc*SBD-UKD. For the *Av*SBD-UKD, the highest fluorescence signal was 35,691, while for *Vc*SBD-UKD, it was 1,366. Like *Vc*SBD-UKD, data from the *Av*SBD-UKD probe show higher signal from glycans with α(1,2) fucose linkages to galactose than the other types of linkages. Also, α(1,3) fucose linkages to *N*-acetylglucosamine were prevalent. Overall, both screening results indicate a similar pattern of glycan recognition between *Av*SBD-UKD and *Vc*SBD-UKD, with the most frequent binding to terminally fucosylated glycans.

### *Vc*SBD-UKD and *Av*SBD-UKD have a strong affinity for l-fucose

**Fig 4:**
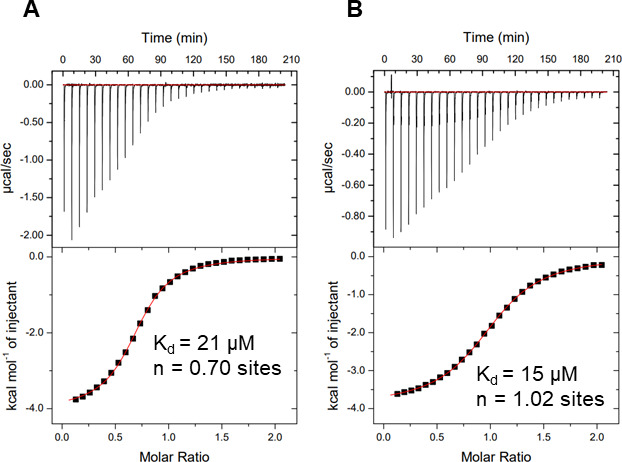
ITC data of SBD-UKD binding to l-fucose. Thermograms of the interaction between l-fucose and *Vc*SBD-UKD (**A**) and *Av*SBD-UKD (**B**). The raw reads over the duration of the titrations (top panel) and fitted curve (bottom panel) are shown, along with the calculated *K*_d_ and stoichiometry values.

To further characterize the sugar-binding function of *Vc*SBD-UKD and *Av*SBD-UKD, isothermal titration calorimetry (ITC) was used to determine the binding affinities and thermodynamic profiles of SBD-UKD interactions with monomeric l-fucose. The titration of l-fucose with each protein produced higher exothermic peaks in the beginning injections and lower heat measurements from later injections. Integration of the raw data using a one-site binding model produced two-sided sigmoidal curves with similar dissociation constants (*K*_d_) in the μM range and slightly different stoichiometry values (*N*). For *Vc*SBD-UKD, calculated ITC values yielded a *K*_d_ of 21 µM and *N* of 0.70 (Fig. 4A). For a single-site binding protein, a stoichiometry value less than 1.0 may indicate a loss of protein function, perhaps through proteolysis, which could potentially affect the accuracy of the resulting *K*_d_ calculation. Indeed, a previous ITC study using the 37 kDa *Vc*SBD-UKD fragment yielded a *K*_d_ of 21 µM and N of 0.30 (data not shown). When ITC was conducted on *Av*SBD-UKD, the thermodynamic measurements yielded a *K*_d_ of 15 µM and a *N* of 1.0 (Fig. 4B). For both sets of data, the c-values, which is a measure of how sigmoidal each curve is, were within the acceptable range for accurate *K*_d_ and *N* calculation.

The binding of fucose to *Vc*SBD-UKD produced a large change in enthalpy (Δ*H* = −4,019 kcal mol^−1^) and a weaker change in entropy (−*T*Δ*S* = −202 kcal mol^−1^) (Table S1). The large negative Δ*H* suggests the formation of many favorable intra- and intermolecular bonds (e.g., hydrogen bonding and van der Waals), and the smaller negative −*T*Δ*S* suggests the formation of favorable hydrophobic interactions. The same pattern was observed with *Av*SBD-UKD, yielding a Δ*H* = −3,897 kcal mol^−1^ and −*T*Δ*S* = −229 kcal mol^−1^. Both interactions produced strong changes in Gibbs free energy (Δ*G*), which indicates the interaction occurs favorably and spontaneously.

### *Vc*SBD-UKD binds and lyses erythrocytes, and hemolysis is inhibited by l-fucose

**Fig 5:**
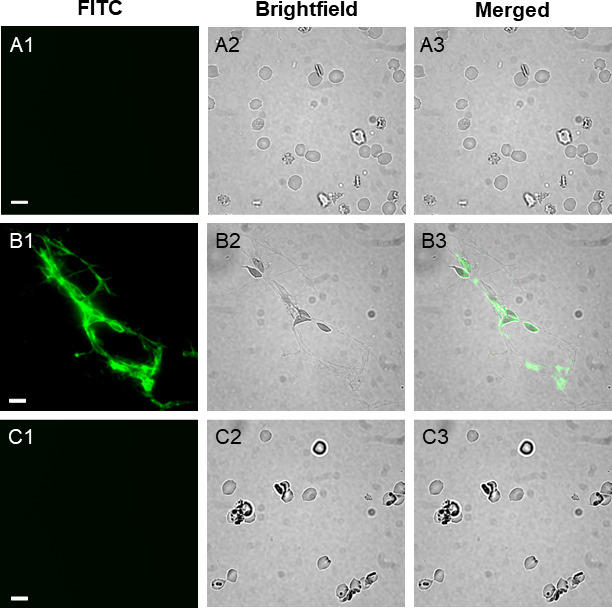
Fluorescence microscopy images of *Vc*SBD-UKD and erythrocytes. Fluorescence images of negative control (row A), erythrocytes treated with *Vc*SBD-UKD (row B), and erythrocytes treated with *Vc*SBD-UKD and 5 mM l-fucose (row C). The columns are annotated with different types of views. The white line is a scale bar representing 10 µm.

As was demonstrated earlier in the glycan array experiments, FITC-labeled *Vc*SBD-UKD bound to fucosylated glycans, many of which are blood group epitopes, which suggests this domain might contribute to FrhA-dependent binding of *V. cholerae* to erythrocytes. To investigate this possibility, FITC-labeled *Vc*SBD-UKD was incubated with erythrocytes for 15 min and observed under a fluorescence microscope. Microscopic images show clumps of distorted and lysed erythrocytes surrounded by cellular debris (Fig. 5 A2 through C2). Fluorescence signal was observed on erythrocyte membranes and cellular debris, which suggests that FITC-labeled *Vc*SBD-UKD can bind and lyse erythrocytes (Fig. 5 A1 through C1). The addition of 5 mM l-fucose to labeled *Vc*SBD-UKD prior to incubation with erythrocytes blocked both binding and lysis (Fig. 5 A3 through C3). The microscopy images showed normal erythrocyte morphology and no visible sign of cell lysis. Also, there was no observable fluorescence signal on the erythrocytes.

**Fig 6:**
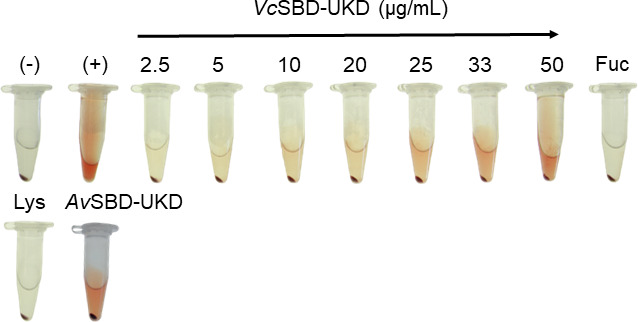
Images of hemolysis assay. Images of erythrocyte aliquots incubated with *Vc*SBD-UKD dilutions. Protein concentration (μg/mL) is indicated by values above sample and under black line. (−) and (+) represent negative and positive controls, respectively. Other treatments are annotated accordingly.

To test the apparent hemolytic activity of *Vc*SBD-UKD, a hemolysis assay was performed using increasing concentrations of purified protein and visual inspection of the erythrocyte sample supernatant for the appearance of red color. The negative controls (blank and lysozyme treatments) had a clear supernatant, while the positive control, which was erythrocytes that had been treated with lysis buffer, was an intense red (Fig. 6). The supernatant of the erythrocytes treated with *Vc*SBD-UKD increased in red color intensity as the concentration of the added protein increased from 2.5 to 50 µg/mL. At the lowest tested concentration, the supernatant was almost colorless, whereas at the highest tested concentration, the supernatant was red. When the ortholog *Av*SBD-UKD was added at 50 µg/mL to erythrocytes, the resulting supernatant was similarly red. When 5 mM l-fucose was preincubated with the highest concentration of *Vc*SBD-UKD prior to addition to erythrocytes, the supernatant remained as colorless as the negative control. In all samples except the positive lysis control, a red pellet was observed at the bottom of the tube after centrifugation.

**Fig 7:**
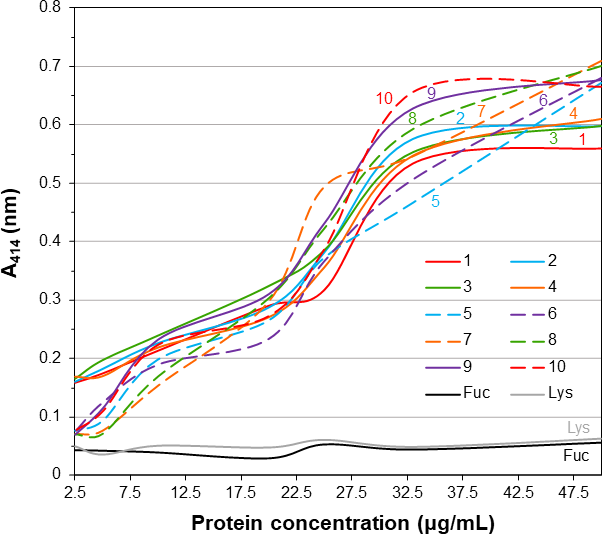
Line graph of hemolysis assay. Absorbance of samples treated with increasing concentrations (μg/mL) of *Vc*SBD-UKD protein was measured at *A*_414_ nm. The *x*-axis and *y*-axis represent protein concentration in µg/mL and absorbance at 414 nm, respectively. In the legend (inset), numbers above lines and text adjacent to colored lines represent replicate number and other treatments.

To quantitatively characterize the degree of hemolysis caused by *Vc*SBD-UKD, the supernatants from the hemolysis assay samples were measured at an absorbance of 414 nm. An increase in *A*_414_ nm is correlated with an increase in cell-free heme-containing proteins, mostly erythrocyte hemoglobin. The lysis supernatants of 10 replicates of increasing protein concentration were measured. As *Vc*SBD-UKD concentration increased, so did the *A*_414_ nm of the supernatant (Fig. 7). The average absorbance of the highest protein concentration replicates was 0.63, while average absorbance of the lowest protein concentration replicates was 0.11. For all *Vc*SBD-UKD replicates, the absorbance lines increased in a non-linear serpentine manner. To ensure hemolysis was not simply induced by the presence of non-specific protein, lysozyme was added at identical concentrations. The *A*_414_ nm of lysozyme did not increase with protein concentration and stayed steady at an average value of 0.051. When 5 mM l-fucose was preincubated with *Vc*SBD-UKD, the *A*_414_ nm also remained at an average value of 0.043.

### Fucose blocks *V. cholerae* binding to Hep-2 cells

**Fig 8:**
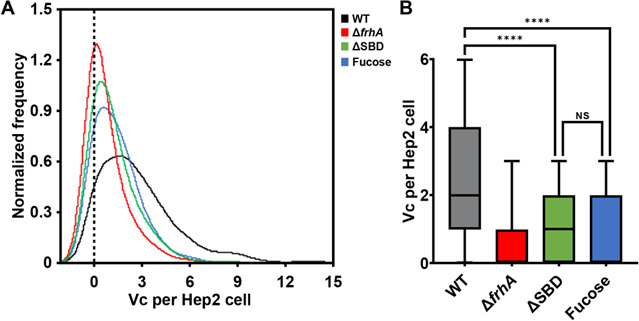
The sugar-binding domain participates in *V. cholerae* binding to Hep-2 cells. (**A**) Average number of wild-type *V. cholerae* binding to a Hep-2 cell is shown by the black line. The blue line shows bacterial binding in the presence of 0.2 mM l-fucose. The red and green lines show the binding of *V. cholerae* deletion constructs lacking FrhA or SBD, respectively. (**B**) Statistical analysis of cell-binding data from (**A**).

*V. cholerae* FhrA facilitates binding to the human epithelial cell line Hep-2 (20, 22). To determine the contribution of *Vc*SBD to *V. cholerae* Hep-2 binding, we measured binding of RFP-tagged wildtype, Δ*frhA*, and Δ*frhA*SBD *V. cholerae* cells in the absence and presence of l-fucose by imaging flow cytometry (Fig. 8). The number of fluorescent *V. cholerae* bacteria bound per cell was quantitated by measuring approximately 1000 Hep-2 cells (Fig. 8A; Table S2). As shown previously (20), *V. cholerae* binds to Hep-2 cells, and removal of the entire FrhA coding sequence results in a ~70% reduction in *V. cholerae* binding (Fig. 8B). Removal of just the SBD from FrhA resulted in ~55% reduction in *V. cholerae* bound to Hep-2 cells. Likewise, the addition of 0.2 mM l-fucose to wild-type *V. cholerae* led to a ~45% reduction in *V. cholerae* bound to Hep-2 cells, which was not significantly different than the ΔSBD *V. cholerae* binding. These results are consistent with FrhA SBD binding to fucosylated residues contributing to *V. cholerae* epithelial cell binding.

### Mapping the domain boundaries of SBD-UKD

**Fig 9:**
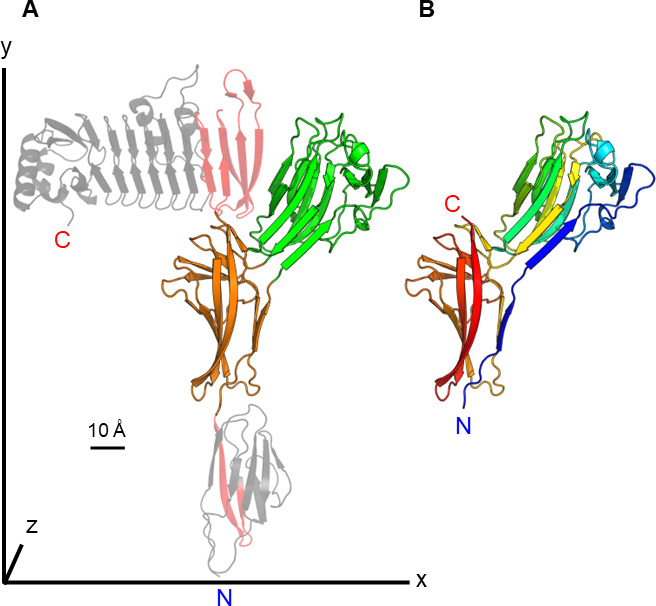
AlphaFold-predicted structure of *V. cholerae* FrhA C-terminal end. (**A**) The structure of the Ig-like domain, SBD, UKD, and RTX β-roll as a continuous protein unit displayed using PyMOL. The SBD and UKD are colored green and orange, respectively. The Ig-like domain (bottom) and RTX β-roll (top) are colored gray. The red color indicates the residues added to the 42 kDa *Vc*SBD-UKD construct that are extra. A scale bar, depicted as a black line, represents 10 Å for reference. (**B**) Structure of the *Vc*SBD-UKD using a rainbow color scheme. The N (blue) and C (red) termini are represented by letters N and C, respectively.

When AlphaFold2 became available, it was used retrospectively to better define the boundaries of the SBD-UKD domains and to predict the structure of *Vc*SBD-UKD in relation to the preceding Ig-like domain and subsequent RTX β-roll. The structure of this FrhA C-terminal end region was then displayed using PyMOL (Fig. 9). The AlphaFold2-predicted structure showed the presence of four folded protein domains (Fig. 9A). The RTX β-roll (colored gray) folded into a typical Ca^2+^-containing β-solenoid (23), while the three other domains adopted β-sandwich folds, as expected. The Ig-like domain at the N-terminal end (gray) and UKD (orange) are organized in a linear fashion along the *y*-axis, while the SBD (green) is projected outward at approximately a 45-degree angle. The RTX β-roll lies linearly parallel to the *x*-axis. The whole protein has dimensions of 104.5 × 46.4 × 141.7 Å. The *Vc*SBD-UKD protein unit measures 46.9 × 43.1 × 78.0 Å. The SBD consists of 11 β-strands, while the UKD contains 9 β-strands organized in an anti-parallel fashion.

When *Vc*SBD-UKD is colored in the rainbow scheme from N terminus (blue) to C terminus (red), it can be observed that the SBD’s N and C termini emerge from the same part of UKD near each other (Fig. 9B). Also, the UKD is split into two uneven sections by the SBD. The first section consists of a single β-strand at the N terminus, and the second section, which is the bulk of the UKD, rejoins the single β-strand after the protein forms the SBD. Looking at the UKD of the AlphaFold2-determined domain map, it is discontinuous in sequence. A short stretch of 12 amino acids starts the UKD at the N terminus before the SBD emerges, and the rest of the 106 amino acids comes after the SBD at the C terminus. In total, the SBD and UKD are 175 and 118 amino acids in length, respectively.

### BioSAXS produced a high-quality molecule envelope of the trimmed *Vc*SBD-UKD

**Fig 10:**
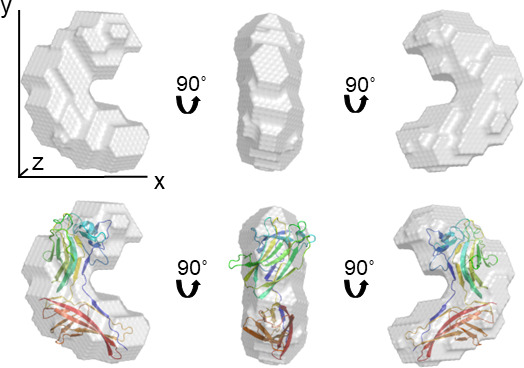
Solution structure of *Vc*SBD-UKD by SAXS. Different orientations of the SAXS molecular envelope are shown rotated 90° along a centered vertical axis. The AlphaFold-predicted structure, colored using rainbow colors, was manually fitted into the SAXS envelop. Axis lines *x*, *y*, and *z* represent the height, width, and length of the envelope.

The Guinier plot of *Vc*SBD-UKD examined by BioSAXS displayed strong linearity, suggestingthat the protein solution was free of protein aggregation and large molecular weight contaminants (Fig. S5). Guinier fit residuals were flat and randomly distributed about zero. Several residuals to the right of the minimum limit located near 0.0000 *q*^2^ were omitted from the fit to allow for stronger linearity. Based on the data from the Guinier plot and calculations performed by the software, the molecular weight of the *Vc*SBD-UKD construct was 38.2 kDa. To obtain a solution structural envelope of the *Vc*SBD-UKD fragment, a low-resolution model was constructed from experimental SAXS data using the *ab initio* modeling program DAMMIF. DAMMIF uses enclosed search volume of densely packed dummy atoms to reconstruct the shape of the protein in solution. The resulting molecular envelope of the protein has an l-shaped fold (Fig. 10), with a height, length, and width of 79.6 Å, 59. 3 Å, and 33.1 Å, respectively. The AlphaFold2 predicted structure of *Vc*SBD is in excellent agreement with the solution structural envelope produced by BioSAXS.

## DISCUSSION

### The role of the SBD-UKD pair in bacterial adhesion

Our findings indicate SBD-UKD of *V. cholerae* and *A. veronii* bind preferentially to H antigens from the ABH blood group and Le^a, x, b, y^ epitopes from the Lewis blood group. The protein did not bind to identical glycan precursor types lacking Fucα1–2, α1–3, and α1–4 Fuc, which indicates that protein-binding interaction occurs to the listed fucose moieties. Binding to α1–6 fucose residues was also observed but at lower RFU values. Coupled with binding observed from immobilized fucose residues on the array, the minimum epitope is most likely fucose though many fucosylated glycans did not bind. This might be explained by the different branching, linkages, and orientations of nearby sugar residues, which may interfere with protein binding. Although the protein from both species of bacteria bound to terminally fucosylated glycans, the recognition patterns were slightly different. This could be due to a difference in glycan density between the two glycan array versions or a subtle difference in the ligand-binding site.

The H epitope is found on all blood types, which may allow the SBD-UKD to bind to any blood type (24). The Lewis epitopes are also found on erythrocytes. The glycan array data showing preference for blood group epitopes and our fluorescence microscopy experiments showing *Vc*SBD-UKD binding to erythrocytes suggest the protein binds to blood group epitopes on erythrocytes. Although *V. cholerae* binds to blood *in vitro*, infection of the blood in affected individuals is quite rare (25, 26). However, these blood group epitopes are also found on epithelial cells, like intestinal cells (27). Consistent with this, *V. cholerae* binding to epithelial cells can be competed with l-fucose, and *V. cholerae* containing a deletion of the SBD is defective at binding epithelial cells, to the same degree that l-fucose inhibits binding. Coupled with the fact *V. cholerae* binds to intestinal cells and previous studies showing the importance of FrhA in intestinal colonization, it is likely that the SBD-UKD of FrhA facilitates the adhesion of *V. cholerae* to intestinal cells as illustrated here with the Hep2 cell-binding experiments. Other potential binding partners for SBD-UKD are mucins in the mucosal layer. The SBD-UKD of FrhA could help localize the bacterium toward the mucosa by using the SBD-UKD of FrhA. The SBD-UKD of *A. veronii* may also be involved in binding to epitopes found in the digestive tract and/or blood.

Many bacterial and viral pathogens produce adhesins that bind to fucosylated glycans in the host intestinal tract. For example, *Helicobacter pylori*, the causative agent of peptic ulcers, uses BabA adhesin to mediate adherence to human gastric epithelial cells bearing fucosylated ABH and/or Le^b^ blood group epitopes (28). Norovirus and rotavirus, which cause gastroenteritis, encode viral adhesins that bind to α(1,2)-fucosylated glycans on A-type blood and Le^x^ epitopes, respectively (29). Another example is *Salmonella enterica*, which produces fimbriae that bind to α(1,2)-fucosylated glycans on epithelial cells (30).

Since the SBD-UKD was shown to bind blood group epitopes, the protein was tested for erythrocyte binding. Fluorescence microscopy images showed it not only bound to erythrocytes, but it also unexpectedly lysed them. This was also demonstrated using quantitative hemolysis assays, which showed an increase absorbance at 414 nm as SBD-UKD concentration increased. Typically, the mechanism of action of hemolysins can either be through pore formation using oligomers (31, 32) or enzymatic digestion of the lipid membrane (33). The mechanism of hemolysis by *Vc*SBD-UKD and *Av*SBD-UKD is unknown. It is possible that the SBD is a localization domain responsible for targeting the protein to the surface of an erythrocyte, where it might be involved in oligomerization. The non-linear dose-response curves observed in the hemolysis assay may reflect the concentration-dependent oligomerization of the protein. Although recombinant SBD-UKD has hemolytic activity, we are unable to attribute the cause of hemolysis to a particular domain and it is not known if the entire FrhA adhesin has hemolytic activity. The fact that *Vc*SBD-UKD behaves as a hemolysin raises the question of whether a secreted RTX toxin of this type might have evolved into an anchored RTX adhesin to secure the bacterium to its host for more prolonged and efficient access to the nutrients in the lysed cells.

### Inhibiting bacterial adhesion by targeting the SBD-UKD using fucosylated ligands

It is known that *V. cholerae* uses FrhA to bind erythrocytes and intestinal cells. With the development of sugar-based inhibitors, saturation of the SBD-UKD could potentially inhibit attachment of *V. cholerae* to host cells, as seen with l-fucose (Fig. 8). For example, several novel compounds like D- and C-mannosides have been found to bind to FimH and inhibit adhesion of uropathogenic *E. coli* to urothelial cells (34, 35). Since the SBD-UKD binds to mammalian fucosylated glycans, it might be possible to use fucose-based analogs to inhibit binding. Interestingly, cholera toxin, which is the signature effector protein of *V. cholerae* that causes cholera disease, has been shown to bind to fucosylated glycans similar to the ones identified in this study (36). Thus, it might be possible to use a fucose derivative to inhibit both bacterial adhesion and cholera toxin binding, to further reduce pathogenesis.

ITC was used to determine the affinity of *Vc*SBD-UKD and *Av*SBD-UKD for binding l-fucose. Results indicate the former and latter have *K*_d_ values of 21 µM and 15 µM, respectively. These binding affinities are similar as might be expected for orthologs. The affinity of a typical lectin for monosaccharides is in the low millimolar range (37). There are several sugar-binding adhesins produced by pathogenic bacteria that bind fucose at a micromolar binding affinity. For example, the adhesin BambL from *Burkholderia ambifaria*, an opportunistic pathogen that infects the lungs of immunocompromised individuals, binds to monomeric l-fucose with an affinity less than 1 µM (38).

As described above, *Av*SBD-UKD had a stoichiometry value of 1.0 for fucose binding, which indicates that the protein has a single sugar-binding site. The highest stoichiometry value for *Vc*SBD-UKD was 0.7. For a single-site binding protein, a stoichiometry value less than 1.0 may indicate an impure protein sample, loss of protein function, and/or a protein concentration that is lower (or ligand concentration higher) than expected (39). Loss of protein function due to proteolysis seems the most likely of these explanations for the stoichiometry of *Vc*SBD-UKD.

Although we have shown strong binding of SBD-UKD to monomeric l-fucose, ITC or other types of quantitative binding studies using complex fucosylated polysaccharides may provide different binding affinities and insight on the binding dynamics of SBD-UKD. It is possible that studies with fucosylated complex glycans identified in the glycan arrays may produce different binding affinities since monomeric l-fucose is not restricted by sugar linkages or sterically hindered by adjacent carbohydrate units found in fucosylated glycans. Binding studies with different fucosylated glycans might demonstrate what types of linkages and adjacent sugar units can enhance or weaken the binding affinity, allowing for rational design or selection of fucose-based inhibitors.

### AlphaFold2 is an invaluable tool for characterizing adhesin domains and their boundaries

Using AlphaFold2, a section of the FrhA ligand-binding region was analyzed to predict the structure of the SBD-UKD unit and map its domain boundaries. The AlphaFold2-predicted structure showed that the UKD adopts a split Ig-like domain fold. Split Ig-like domains have previously been seen in RTX adhesins (12). They are similar in fold to BIg domains; however, unlike BIg domains, they are discontinuous in primary amino acid sequence. Since they are discontinuous in sequence and not well-characterized as a type of domain, homology-dependent programs may not be able to identify them. Thus, without an experimental or AlphaFold2-predicted structure, split Ig-like domains are difficult to recognize and map.

To date, split Ig-like domains have not been shown to bind ligands, leading to the plausible assignment of the fucose-binding function to the SBD. Moreover, by discovering the identity of the UKD, it excludes its role as another ligand-binding domain responsible for any of the other binding functions of FrhA. The UKD might be involved indirectly in ligand binding by helping the SBD adopt specific outward orientations to improve ligand accessibility. *Mp*IBP and FrhA contain split Ig-like domains, which appear to orient the PA14 and PBD domains away from the rest of the protein, respectively (12, 40). By using split domains, the N and C termini of the ligand-binding domain are on one side, reducing steric hindrance around the binding pocket located on the opposite end of the domain.

In addition to recognizing the UKD as a split Ig-like domain, AlphaFold2 assisted in mapping the domain boundaries of the split Ig-like domain and SBD. Bacterial RTX adhesins are unusual in being extremely long, continuous polypeptide chains that fold into many different domains joined end to end. The most varied section of these adhesins is the ligand-binding region. To study the ligand-binding domains of RTX adhesins structurally and functionally, they must be mapped out accurately. Without defining their termini, protein constructs missing essential parts of domains may not be able to fold properly, leading to unstable, insoluble, and/or non-functional proteins, which defy characterization. The AlphaFold2-predicted structure revealed that the split Ig-like domain was about 7 kDa shorter on the C-terminal end than previously predicted. In other words, the 42 kDa *Vc*SBD-UKD had an additional protein mass of 7 kDa belonging to the subsequent domain, which was the RTX β-roll as is indicated by in red (Fig. 9A). Without the full RTX β-roll sequence, it is unlikely that the 7 kDa partial domain can fold properly and is consequently degraded. This may explain the emergence of the 37 kDa fragment observed during and after purification, and the apparent degradation of FrhA observed when a deletion of UKD extended into this β-roll (20). Additional evidence includes the lack of extra protein mass in the experimentally determined molecular envelope and the fitting of the AlphaFold2-predicted structure in the BioSAXS structure.

## MATERIALS AND METHODS

### Molecular cloning of *Vc*SBD-UKD, *Av*SBD-UKD, and *Av*GFP-SBD-UKD genes

The gene encoding the *Av*SBD-UKD construct was synthesized (GenScript), and the DNA construct of *Vc*SBD-UKD was PCR amplified from synthesized FrhA ligand-binding region +T1 SS region gene (GeneArt). Both constructs were cloned into a pET-28a vector with optimal codon usage for protein expression in *E. coli*. The DNA constructs were bounded by *Nde*I and *Xho*I restriction sites at the 5ʹ and 3ʹ ends, respectively. To produce the GFP-tagged version of the *Av*GFP-SBD-UKD construct, a GFP gene with *Nde*I restriction sites was PCR amplified from a pCR 2.1 vector and inserted into the *Av*SBD-UKD clone cut by single-site digestion using *Nde*I. Plasmids were transformed into chemically competent TOP10 cells for purification, and validation by DNA sequencing (Robarts Research Institute), prior to electroporation into BL21(DE3) cells for protein expression.

### Expression and purification of *Vc*SBD-UKD, *Av*SBD-UKD, and *Av*GFP-SBD-UKD

To express *Vc*SBD-UKD and *Av*SBD-UKD, single colonies of BL21 (DE3) *E. coli* cells were inoculated into 25 mL cultures of LB broth with 0.1 mg/mL kanamycin and grown at 37°C for 16 h. Overnight cultures were used to inoculate 1 L cultures containing 0.1 mg/mL kanamycin, which were grown until an OD_600_ of 0.85 was reached. IPTG was then added to a final concentration of 1 mM to induce protein production at 23°C overnight for 16 h. For *Av*GFP-SBD-UKD, single colonies of ArcticExpress (DE3) *E. coli* cells were grown overnight as described earlier with the addition of 0.1 mg/mL of ampicillin during the inoculation and growth steps. IPTG was then added to a final concentration of 0.1 mM to induce protein at 10–13°C for 24 h.

Protein-expressing *E. coli* cultures were centrifuged at 4500×*g* in a JS-4.2 rotor (Beckman Coulter). The supernatant was discarded, and the cell pellet resuspended in 25 mL of Ni buffer (50 mM Tris-HCl pH 7.6, 500 mM NaCl, 10 mM imidazole, 5 mM CaCl_2_) along with a protease inhibitor cocktail tablet (Roche). Cells were then lysed by sonication and the resulting cell lysate centrifuged at 30,000×*g* in a JA-25.5 rotor (Beckman Coulter) to separate supernatant from cell debris. The lysate supernatant was incubated with Ni-NTA Agarose Resin (Qiagen) in a beaker with 150 mL of Ni buffer; the resin was separated from the top liquid layer and then loaded on the column. The resin was then washed with three column volumes of Wash buffer (50 mM Tris-HCl pH 7.6, 500 mM NaCl, 30 mM imidazole, 5 mM CaCl_2_). The bound protein was eluted using Elution buffer (50 mM Tris-HCl pH 7.6, 500 mM NaCl, 400 mM imidazole, 5 mM CaCl_2_). Eluted fractions were pooled and subjected to anion exchange chromatography on a HiLoad 16/10 Q Sepharose column (GE Healthcare). The column was equilibrated with Buffer A (50 mM Tris-HCl pH 7.6 and 5 mM CaCl_2_) and protein eluted using a linear NaCl gradient from 0 to 1 M. Eluted fractions were pooled, concentrated to 5.0 mL, and subjected to size-exclusion chromatography on a HiLoad 16/60 Superdex 75 column (GE Healthcare) using SEC buffer (20 mM Tris-HCl pH 7.6, 200 mM NaCl, 5 mM CaCl_2_) at a flow rate of 1.5 mL/min. Fractions (3.0 mL) were collected. Protein purity and yield were assessed using SDS-PAGE and UV/Vis spectroscopy, respectively.

### Mammalian glycan array screening of labeled *Vc*SBD-UKD and *Av*GFP-SBD-UKD

Purified 37 kDa *Vc*SB-UKD fragment was fluorescently labeled by primary amine conjugation using fluorescein isothiocyanate (FITC) (Thermo Fisher Scientific). A sample with 2 mg of protein in 1 mL of SEC buffer was dialysed overnight for 16 h in 20 mM HEPES (pH 8.0), 100 mM NaCl, and 2 mM CaCl_2_ to remove the primary amine Tris that would otherwise have reacted with fluorescein. While stirring, 0.5 mg of fluorescent dye suspended in 0.05 mL of DMSO was added to the dialyzed protein sample and incubated in the dark at 4°C for 4 h. Unbound dye was separated from the protein conjugate by size-exclusion chromatography on an 8.3 mL Sephadex G-25 M column equilibrated in SEC buffer. FITC-labeled *Vc*SBD-UKD (1 mg/mL) and GFP-tagged *Av*SBD-UKD (1.32 mg/mL) protein were sent to the CFG for analysis against the mammalian glycan screen v. 5.2 and v. 5.5, respectively. In brief, samples were detected on the glycan array, which consists of glycans immobilized on a glass plate using *N*-hydroxysuccinimide esters, by fluorescence after incubation with 70 µL of probe at a protein concentration of 50 µg/mL for 1 h. After incubation, the plates were washed three times to remove non-specific binding and dried under nitrogen before scanning using a fluorometer. The data were reported as an average of four replicates after removing the lowest and highest point from each set to avoid spurious values. The complete data set is freely available through the CFG website (http://www.functionalglycomics.org/static/consortium/consortium.shtml).

### Isothermal titration calorimetry of *Vc*SBD-UKD and *Av*SBD-UKD to l-fucose

Samples containing a mixture of 42 and 37 kDa *Vc*SBD-UKD (524 µM) and *Av*SBD-UKD (230 µM) were dialyzed against SEC buffer overnight for 16 h using a 6–8 kDa cut-off cellulose acetate membrane. Solutions of 5,240 and 2,300 µM l-fucose were made in the same buffer. Ligand (1.3 µL) was titrated into 350 µL of dialyzed protein by computer-controlled syringe at 7 min intervals for a total of 29 injections at 25°C. ITC data were collected using a MicroCal iTC200 calorimeter and analyzed using Origin software Version 7.0.

### Hemolysis assay of *Vc*SBD-UKD and *Av*SBD-UKD

Diluted stocks of a mixture of 42 and 37 kDa *Vc*SBD-UKD and *Av*SBD-UKD were added to 2% O type blood suspension in KRT buffer (120 mM NaCl, 5 mM KCl, 1 mM MgSO_4_, 3 mM CaCl_2_, 10 mM Tris-HCl pH 7.4). Blood was obtained from Zen-Bio (3920 S Alston Ave, Durham, NC 27713). For the positive control, 50 µL of RIPA Lysis Buffer (Thermo Fisher Scientific) was added to a blood sample in place of *Vc*SBD-UKD. Samples were left to incubate on a shaker for 15 min at room temperature. For ligand inhibition samples, 5 mM l-fucose was added to the highest concentrated protein sample for 5 min before the 15 min incubation. After incubation, samples were spun down to separate supernatant from cell debris for 3 min at 9.6×*g* in an accuSpin Micro 17 rotor (Thermo Fisher Scientific). Images of the hemolysis assay tubes were taken using a Samsung Galaxy S20 +Android smartphone. Supernatant absorbance measurements at 414 nm were recorded and plotted using Microsoft Excel.

### Fluorescence microscopy of labeled *VcS*BD-UKD

A mixture of 42 and 37 kDa *Vc*SBD-UKD was labeled with fluorescein isothiocyanate (FITC) as previously described (11). Labeled protein was incubated with 2% blood for 15 min and washed using KRT buffer by spinning for 3 min at 9.6×*g*. The pellet was washed five times in this way.

Light and fluorescence microscopy imaging were performed using an Andor Zyla 4.2 Plus camera paired with an Olympus IX83 inverted fluorescence microscope. For FITC visualization, an excitation wavelength of 488 nm was used. Images of erythrocytes were captured on brightfield, and TRITC channels separately.

### BioSAXS analysis of *Vc*SBD-UKD

Synchrotron X-ray scattering data were collected at the ID7A1 beamline of the Cornell High Energy Synchrotron Source at Cornell University. Purified 37 kDa *Vc*SBD-UKD fragment sample was run on an in-line S75 size-exclusion column with a resin bed volume of 1.6 × 60 cm at 4°C and SAXS data collected from the highest absorbance fractions. The beamline operated at 7–14 keV. All SAXS data processing steps were performed using BioXTAS RAW version 2.1.4. Molecular shape reconstruction was performed using simulated annealing methods implemented in DAMMIF. Reconstructions were visualized in PyMOL (Schrödinger, LLC).

### Structural prediction of *Vc*SBD-UKD by Alphafold2

Models of proteins were carried out using the monomer option of ColabFold. This notebook (https://github.com/sokrypton/ColabFold) uses multiple sequence alignments produced by MMseqs2 and HHsearch servers as input for template-free structure prediction by AlphaFold2. Structures were presented using PyMOL.

### Image flow cytometry

Hep-2 cells (10^6^) were seeded in DMEM +5% FBS into a 24-well plate and allowed to adhere at 37°C and 5% CO_2_ for 2 h. *V. cholerae* strains KKV598 (WT), Fy_VC_12114 (Δ*frhA*), and KKV2985 (frhA^ΔSBD^) (20) expressing RFP were grown overnight at 37°C in Luria Broth with 100 mg/mL streptomycin and 2 mg/mL chloramphenicol. The cells were then back diluted and grown to mid-log at 37°C for 4 h. 5 × 10^7^ CFU of bacteria (MOI 50:1) were added to Hep-2 cells, and plates were spun at 150×*g* for 5 min. The medium was replaced with KRT buffer, containing the desired concentration of inhibitor, if applicable, and allowed to incubate at 37°C and 5% CO_2_ for 1 h. The plates were spun at 150×*g* for 5 min and washed with KRT buffer three times to remove any unbound bacteria. The cells were then fixed with 4% paraformaldehyde and filtered twice for single cells using 20 µm cell-straining capped tubes (Falcon).

Cells were then imaged using an Amnis ImageStreamX MKII (Millipore, Burlington, MA) image flow cytometer with a 7 µm core at low flow rate and high sensitivity using INSPIRE software. RFP was excited using a 561 nm laser and image data were collected through a 60× objective. Single cells were gated using Area/Aspect Ratio 200–1,000/0.6–1 and 10,000 instances were collected per sample. Images were analyzed using IDEAS software version 6.2 (Millipore, Burlington, MA). Machine learning was applied to count the number of RFP spots present on each cell using the spot count wizard, and histograms were derived using the histogram smoothing operation.

## SUPPLEMENTAL MATERIAL

Fig. S1 mbio.02291-23-s0001.TIF Sequence alignment of *Vc*SBD-UKD constructs.

Fig. S2 mbio.02291-23-s0002.TIF Purification of the *Vc*SBD-UKD construct.

Fig. S3 mbio.02291-23-s0003.TIF *Vc*SBD-UKD proteolysis experiment.

Fig. S4 mbio.02291-23-s0004.TIF Purification of the *Av*SBD-UKD construct.

Fig. S5 mbio.02291-23-s0005.TIF SAXS profiles of *Vc*SBD-UKD fragment.

Legends mbio.02291-23-s0006.docx Supplemental figure and table legends.

Table S1 mbio.02291-23-s0007.TIF Thermodynamic parameters of the binding interactions between the proteins and fucose.

Table S2 mbio.02291-23-s0008.TIF *V. cholerae* binding to Hep-2 cells.

ASM does not own the copyrights to Supplemental Material that may be linked to, or accessed through, an article. The authors have granted ASM a non-exclusive, world-wide license to publish the Supplemental Material files. Please contact the corresponding author directly for reuse.

## References

[1] Krachler AM, Orth K. 2013. Targeting the bacteria-host interface: strategies in anti-adhesion therapy. Virulence 4:284–294. doi:10.4161/viru.24606 23799663 PMC3710331

[2] Satchell KJF. 2011. Structure and function of MARTX toxins and other large repetitive RTX proteins. Annu Rev Microbiol 65:71–90. doi:10.1146/annurev-micro-090110-102943 21639783

[3] Guo S, Vance TDR, Stevens CA, Voets IK, Davies PL. 2019. RTX adhesins are key bacterial surface megaproteins in the formation of biofilms. Trends Microbiol 27:470. doi:10.1016/j.tim.2019.02.001 30826181

[4] Benz R. 2020. RTX-toxins. Toxins (Basel) 12:359. doi:10.3390/toxins12060359 32486155 PMC7354457

[5] Linhartová I, Bumba L, Mašín J, Basler M, Osička R, Kamanová J, Procházková K, Adkins I, Hejnová-Holubová J, Sadílková L, Morová J, Sebo P. 2010. RTX proteins: a highly diverse family secreted by a common mechanism. FEMS Microbiol Rev 34:1076–1112. doi:10.1111/j.1574-6976.2010.00231.x 20528947 PMC3034196

[6] Baumann U. 2019. Structure-function relationships of the repeat domains of RTX toxins. Toxins (Basel) 11:657. doi:10.3390/toxins11110657 31718085 PMC6891781

[7] Guo S, Langelaan DN, Phippen SW, Smith SP, Voets IK, Davies PL. 2018. Conserved structural features anchor biofilm-associated RTX-adhesins to the outer membrane of bacteria. FEBS J 285:1812–1826. doi:10.1111/febs.14441 29575515

[8] Smith TJ, Sondermann H, O’Toole GA. 2018. Type 1 does the two-step: type 1 secretion substrates with a functional periplasmic intermediate. J Bacteriol 200:e00168-18. doi:10.1128/JB.00168-18 29866808 PMC6112007

[9] Guo S, Stevens CA, Vance TDR, Olijve LLC, Graham LA, Campbell RL, Yazdi SR, Escobedo C, Bar-Dolev M, Yashunsky V, Braslavsky I, Langelaan DN, Smith SP, Allingham JS, Voets IK, Davies PL. 2017. Structure of a 1.5-MDA Adhesin that binds its Antarctic bacterium to diatoms and ice. Sci Adv 3:e1701440. doi:10.1126/sciadv.1701440 28808685 PMC5550230

[10] Bar Dolev M, Bernheim R, Guo S, Davies PL, Braslavsky I. 2016. Putting life on ice: bacteria that bind to frozen water. J R Soc Interface 13:20160210. doi:10.1098/rsif.2016.0210 27534698 PMC5014055

[11] Guo S, Vance TDR, Zahiri H, Eves R, Stevens C, Hehemann J-H, Vidal-Melgosa S, Davies PL, Parsek MR. 2021. Structural basis of ligand selectivity by a bacterial adhesin lectin involved in multispecies biofilm formation. mBio 12:e00130-21. doi:10.1128/mBio.00130-21 33824212 PMC8092209

[12] Guo S, Zahiri H, Stevens C, Spaanderman DC, Milroy LG, Ottmann C, Brunsveld L, Voets IK, Davies PL. 2021. Molecular basis for inhibition of adhesin-mediated bacterial-host interactions through a peptide-binding domain. Cell Rep 37:110002. doi:10.1016/j.celrep.2021.110002 34788627

[13] Tasiemski A, Massol F, Cuvillier-Hot V, Boidin-Wichlacz C, Roger E, Rodet F, Fournier I, Thomas F, Salzet M. 2015. Reciprocal immune benefit based on complementary production of antibiotics by the leech Hirudo verbana and its gut symbiont Aeromonas veronii. Sci Rep 5:17498. doi:10.1038/srep17498 26635240 PMC4669451

[14] Syed KA, Beyhan S, Correa N, Queen J, Liu J, Peng F, Satchell KJF, Yildiz F, Klose KE. 2009. The Vibrio cholerae flagellar regulatory hierarchy controls expression of virulence factors. J Bacteriol 191:6555–6570. doi:10.1128/JB.00949-09 19717600 PMC2795290

[15] Mekalanos JJ, Swartz DJ, Pearson GD, Harford N, Groyne F, de Wilde M. 1983. Cholera toxin genes: nucleotide sequence, deletion analysis and vaccine development. Nature 306:551–557. doi:10.1038/306551a0 6646234

[16] Pukatzki S, Provenzano D. 2013. Vibrio cholerae as a predator: lessons from evolutionary principles. Front Microbiol 4:384. doi:10.3389/fmicb.2013.00384 24368907 PMC3857921

[17] Conner JG, Teschler JK, Jones CJ, Yildiz FH. 2016. Staying alive: Vibrio cholerae's cycle of environmental survival, transmission, and dissemination. Microbiol Spectr 4. doi:10.1128/microbiolspec.VMBF-0015-2015 PMC4888910 27227302

[18] Almagro-Moreno S, Taylor RK. 2013. Cholera: environmental reservoirs and impact on disease transmission. Microbiol Spectr 1. doi:10.1128/microbiolspec.OH-0003-2012 26184966

[19] Teschler JK, Zamorano-Sánchez D, Utada AS, Warner CJA, Wong GCL, Linington RG, Yildiz FH. 2015. Living in the matrix: assembly and control of Vibrio cholerae biofilms. Nat Rev Microbiol 13:255–268. doi:10.1038/nrmicro3433 25895940 PMC4437738

[20] Lloyd CJ, Guo S, Kinrade B, Zahiri H, Eves R, Ali SK, Yildiz F, Voets IK, Davies PL, Klose KE. 2023. A peptide-binding domain shared with an antarctic bacterium facilitates Vibrio cholerae human cell binding and intestinal colonization. Proc Natl Acad Sci U S A 120:e2308238120. doi:10.1073/pnas.2308238120 37729203 PMC10523503

[21] Jumper J, Evans R, Pritzel A, Green T, Figurnov M, Ronneberger O, Tunyasuvunakool K, Bates R, Žídek A, Potapenko A, et al.. 2021. Highly accurate protein structure prediction with alphafold. Nature 596:583–589. doi:10.1038/s41586-021-03819-2 34265844 PMC8371605

[22] Gardel CL, Mekalanos JJ. 1996. Alterations in Vibrio cholerae motility phenotypes correlate with changes in virulence factor expression. Infect Immun 64:2246–2255. doi:10.1128/iai.64.6.2246-2255.1996 8675334 PMC174063

[23] Baumann U, Wu S, Flaherty KM, McKay DB. 1993. Three-dimensional structure of the alkaline protease of Pseudomonas aeruginosa: a two-domain protein with a calcium binding parallel beta roll motif. EMBO J 12:3357–3364. doi:10.1002/j.1460-2075.1993.tb06009.x 8253063 PMC413609

[24] Stanley P, Wuhrer M, Lauc G, Stowell SR, Cummings RD. 2022. Structures common to different Glycans, p 165–184. In Varki A RD, Esko JD, Stanley P, Hart GW, Aebi M (ed), Essentials of glycobiology, 4th ed. Cold Spring Harbor. 35536943

[25] Phetsouvanh R, Nakatsu M, Arakawa E, Davong V, Vongsouvath M, Lattana O, Moore CE, Nakamura S, Newton PN. 2008. Fatal bacteremia due to immotile Vibrio cholerae serogroup o21 in vientiane, laos - a case report. Ann Clin Microbiol Antimicrob 7:10. doi:10.1186/1476-0711-7-10 18439249 PMC2373308

[26] Daniel D, Kumar S. 2015. Rare strain of Vibrio cholerae septicemia in a patient with multiple myeloma. Case Rep Crit Care 2015:596906. doi:10.1155/2015/596906 26257967 PMC4518176

[27] Ewald DR, Sumner SCJ. 2018. Human microbiota, blood group antigens, and disease. Wiley Interdiscip Rev Syst Biol Med 10:e1413. doi:10.1002/wsbm.1413 29316320 PMC5902424

[28] Ansari S, Yamaoka Y. 2017. Helicobacter pylori baba in adaptation for gastric colonization. World J Gastroenterol 23:4158–4169. doi:10.3748/wjg.v23.i23.4158 28694656 PMC5483490

[29] Huang P, Xia M, Tan M, Zhong W, Wei C, Wang L, Morrow A, Jiang X. 2012. Spike protein VP8* of human rotavirus recognizes histo-blood group antigens in a type-specific manner. J Virol 86:4833–4843. doi:10.1128/JVI.05507-11 22345472 PMC3347384

[30] Suwandi A, Galeev A, Riedel R, Sharma S, Seeger K, Sterzenbach T, García Pastor L, Boyle EC, Gal-Mor O, Hensel M, Casadesús J, Baines JF, Grassl GA. 2019. Std fimbriae-fucose interaction increases Salmonella-induced intestinal inflammation and prolongs colonization. PLoS Pathog 15:e1007915. doi:10.1371/journal.ppat.1007915 31329635 PMC6675130

[31] Song L, Hobaugh MR, Shustak C, Cheley S, Bayley H, Gouaux JE. 1996. Structure of staphylococcal alpha-hemolysin, a heptameric transmembrane pore. Science 274:1859–1866. doi:10.1126/science.274.5294.1859 8943190

[32] Sugawara T, Yamashita D, Kato K, Peng Z, Ueda J, Kaneko J, Kamio Y, Tanaka Y, Yao M. 2015. Structural basis for pore-forming mechanism of staphylococcal alpha-hemolysin. Toxicon 108:226–231. doi:10.1016/j.toxicon.2015.09.033 26428390

[33] Monturiol-Gross L, Villalta-Romero F, Flores-Díaz M, Alape-Girón A. 2021. Bacterial phospholipases C with dual activity: phosphatidylcholinesterase and sphingomyelinase. FEBS Open Bio 11:3262–3275. doi:10.1002/2211-5463.13320 PMC8634861 34709730

[34] Mydock-McGrane L, Cusumano Z, Han Z, Binkley J, Kostakioti M, Hannan T, Pinkner JS, Klein R, Kalas V, Crowley J, Rath NP, Hultgren SJ, Janetka JW. 2016. Antivirulence c-mannosides as antibiotic-sparing, oral therapeutics for urinary tract infections. J Med Chem 59:9390–9408. doi:10.1021/acs.jmedchem.6b00948 27689912 PMC5087331

[35] Montes-Robledo A, Baldiris-Avila R, Galindo JF. 2021. D-Mannoside FimH inhibitors as non-antibiotic alternatives for uropathogenic Escherichia coli. Antibiotics (Basel) 10:1072. doi:10.3390/antibiotics10091072 34572654 PMC8465801

[36] Wands AM, Fujita A, McCombs JE, Cervin J, Dedic B, Rodriguez AC, Nischan N, Bond MR, Mettlen M, Trudgian DC, Lemoff A, Quiding-Järbrink M, Gustavsson B, Steentoft C, Clausen H, Mirzaei H, Teneberg S, Yrlid U, Kohler JJ. 2015. Fucosylation and protein glycosylation create functional receptors for cholera toxin. Elife 4:e09545. doi:10.7554/eLife.09545 26512888 PMC4686427

[37] Cummings RD, Etzler M, Hahn MG, Darvill A, Godula K, Woods RJ, Mahal LK. 2022. Glycan-recognizing probes as tools, p 645–662. In Varki A (ed), Essentials of Glycobiology, 4th ed. Cold Spring Harbor.

[38] Audfray A, Claudinon J, Abounit S, Ruvoën-Clouet N, Larson G, Smith DF, Wimmerová M, Le Pendu J, Römer W, Varrot A, Imberty A. 2012. Fucose-binding lectin from opportunistic pathogen burkholderia ambifaria binds to both plant and human oligosaccharidic epitopes. J Biol Chem 287:4335–4347. doi:10.1074/jbc.M111.314831 22170069 PMC3281725

[39] Freyer MW, Lewis EA. 2008. Isothermal titration calorimetry: experimental design, data analysis, and probing macromolecule/ligand binding and kinetic interactions. Methods Cell Biol 84:79–113. doi:10.1016/S0091-679X(07)84004-0 17964929

[40] Vance TDR, Guo S, Assaie-Ardakany S, Conroy B, Davies PL. 2019. Correction: structure and functional analysis of a bacterial adhesin sugar-binding domain. PLoS One 14:e0221101. doi:10.1371/journal.pone.0221101 31393952 PMC6687154

