# Supplementary figures and images for: Sugar-binding and split domain combinations in repeats-in-toxin adhesins from Vibrio cholerae and Aeromonas veronii mediate cell-surface recognition and hemolytic activities
Source: mBio. 2024 Jan 3;15(2):e02291-23. doi: 10.1128/mbio.02291-23 (PMC10865825; doi:10.1128/mbio.02291-23)

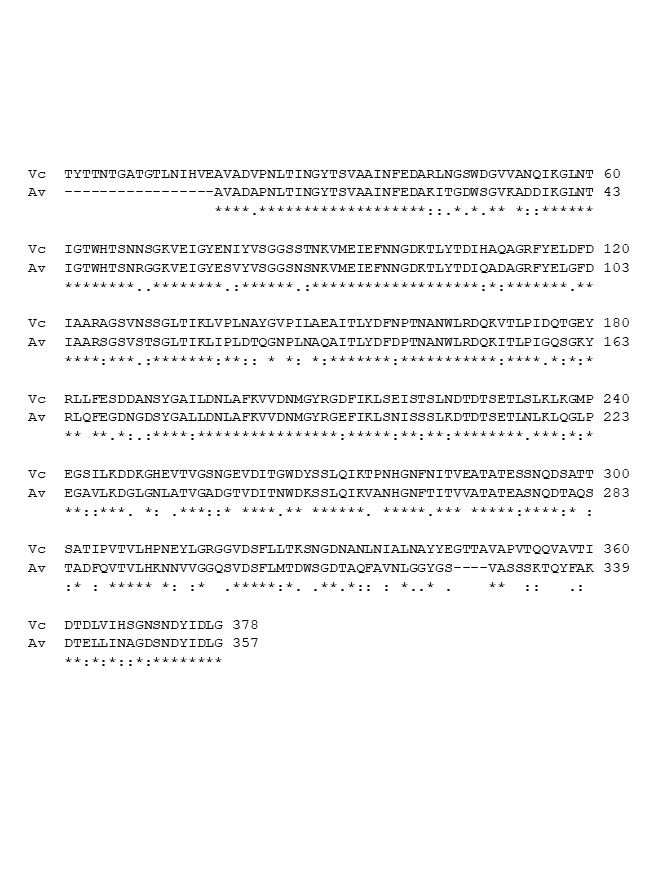

Supplement: Fig. S1 — Sequence alignment of VcSBD-UKD constructs. [file mbio.02291-23-s0001.tif]

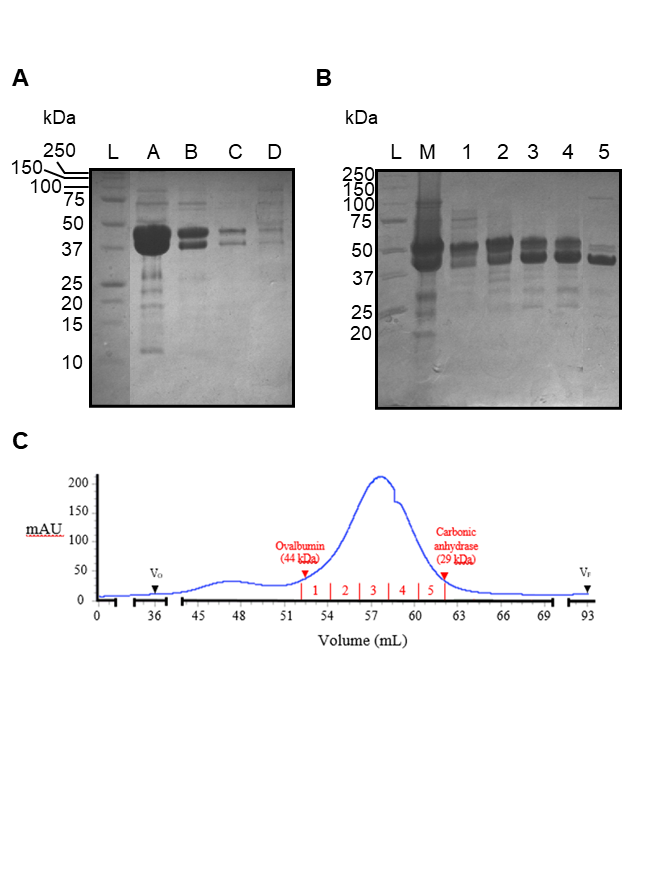

Supplement: Fig. S2 — Purification of the VcSBD-UKD construct. [file mbio.02291-23-s0002.tif]

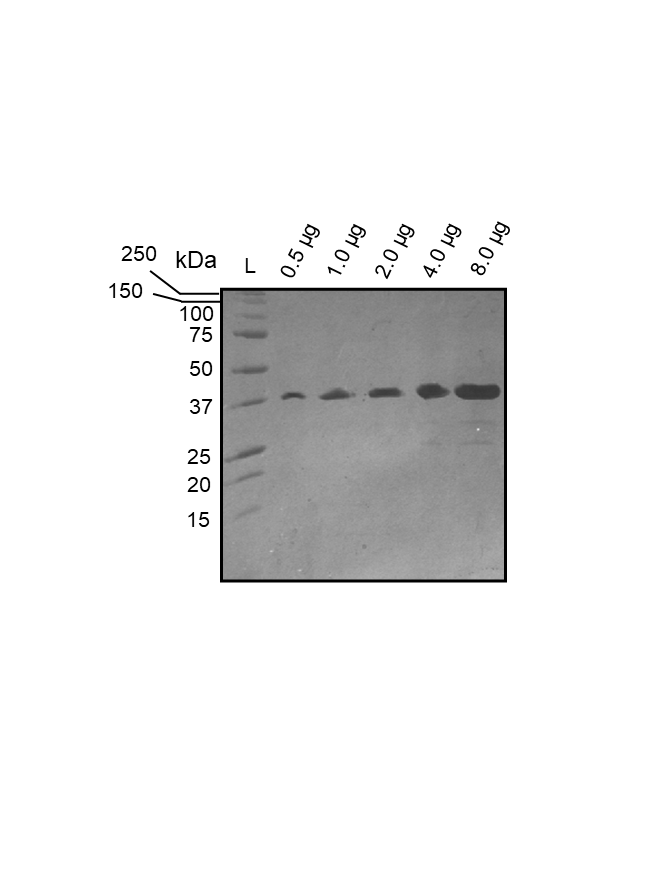

Supplement: Fig. S3 — VcSBD-UKD proteolysis experiment. [file mbio.02291-23-s0003.tif]

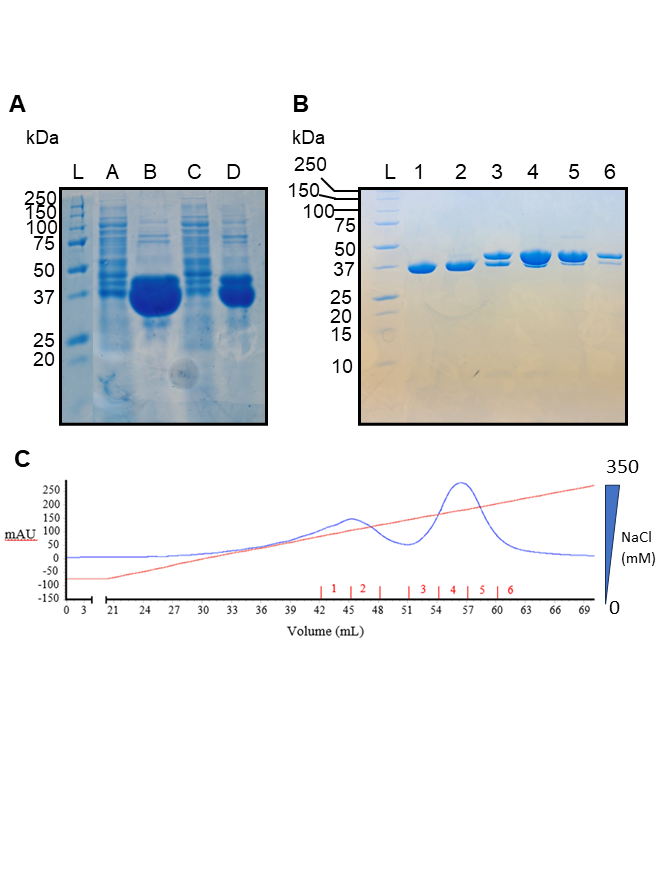

Supplement: Fig. S4 — Purification of the AvSBD-UKD construct. [file mbio.02291-23-s0004.tif]

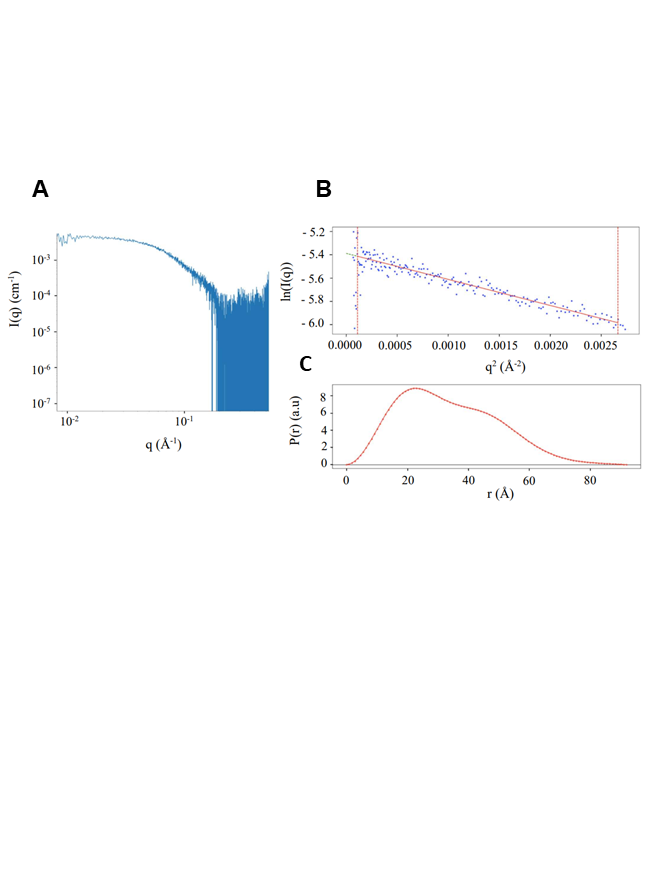

Supplement: Fig. S5 — SAXS profiles of VcSBD-UKD fragment. [file mbio.02291-23-s0005.tif]

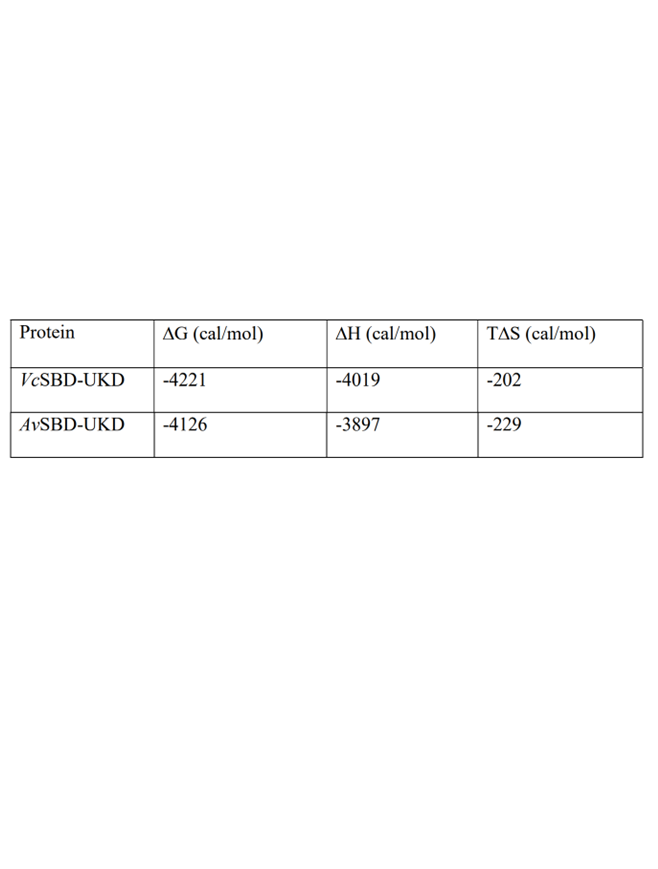

Supplement: Table S1 — Thermodynamic parameters of the binding interactions between the proteins and fucose. [file mbio.02291-23-s0007.tif]

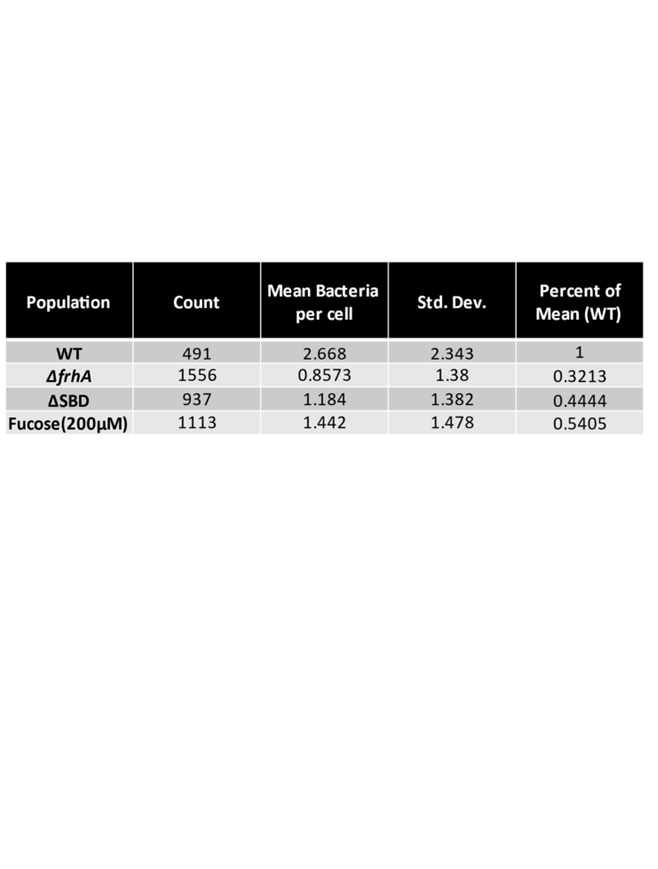

Supplement: Table S2 — V. cholerae binding to Hep-2 cells. [file mbio.02291-23-s0008.tif]
