# Supplementary material for: Sugar-binding and split domain combinations in repeats-in-toxin adhesins from Vibrio cholerae and Aeromonas veronii mediate cell-surface recognition and hemolytic activities
Source: mBio. 2024 Jan 3;15(2):e02291-23. doi: 10.1128/mbio.02291-23 (PMC10865825; doi:10.1128/mbio.02291-23)
Supplement: Legends — Supplemental figure and table legends. [file mbio.02291-23-s0006.docx]

**Figure S1. Sequence alignment of SBD-UKD constructs.** Protein sequences of the SBD-UKD constructs from both bacteria were aligned using Clustal Omega. Residue conservation is indicated as follows: * = identical, : = conservative, . = semi-conservative, space = non-conservative, - = gap. Residue numbers are located at the end of each line of protein sequence.

**Figure S2. Purification of the *Vc*SBD-UKD construct.** SDS-PAGE of Ni-NTA (A), and SEC (B) purification steps. A ladder with molecular weight in kDa is to the left of each gel labeled L. Lanes A, B, C, D, and M represent elution 1, elution 2, wash 1, wash 2, and pooled material from Ni-NTA, respectively. Lanes 1-5 represent fractions in the bottom chromatogram bordered by vertical red lines. (C) SEC chromatogram of pooled fractions from Ni-NTA. The absorbance (y-axis) was measured over a single column volume of 93 mL (x-axis). Void volume (V_O_) and total volume (V_T_) are represented by black triangles. Molecular weight standards are represented by red triangles (X = ovalbumin 44 kDa, Y = Carbonic anhydrase 29 kDa).

**Figure S3. *Vc*SBD-UKD proteolysis experiment.** SDS-PAGE of *Vc*SBD-UKD after a month in the fridge at 4˚C. Increasing amounts of protein were added and labeled above each lane. A ladder with molecular weight in kDa is to the left of the gel.

**Figure S4. Purification of the *Av*SBD-UKD construct.** SDS-PAGE of Ni-NTA (A) and AEX (B) purification steps. A ladder with molecular weight in kDa is to the left of each gel labeled L. Lanes A, B, C, and D represent elution 1, elution 2, wash 1, and wash 2. Lanes 1-6 represent fractions in the bottom chromatogram bordered by vertical red lines. (C) AEX chromatogram of the elution fraction from Ni-NTA. The absorbance (y-axis) was measured over a single column volume of 69 mL (x-axis). The red line represents an increasing salt gradient.

**Figure S5. SAXS profiles of *Vc*SBD-UKD fragment.** (A) Buffer-subtracted SAXS plot on a log-log scale. (B) Guinier plot with a monodisperse residuals (blue line) and fit line (red line). (C) distance distribution P(r) function plot. Data processing and resulting plots were produced using BioXTAS RAW software.

**Table S1.** Thermodynamic parameters (cal mol-1) of the binding interactions between the proteins and fucose.

**Table S2.** *V. cholerae* binding to Hep-2 cells. Statistical analysis of Figure 8.
